# A hybrid machine-learning approach for analysis of methane hydrate formation dynamics in porous media with synchrotron CT imaging

**DOI:** 10.1107/S1600577523005635

**Published:** 2023-07-19

**Authors:** Mikhail I. Fokin, Viktor V. Nikitin, Anton A. Duchkov

**Affiliations:** a Institute of Petroleum Geology and Geophysics SB RAS, 630090 Novosibirsk, Russia; b Advanced Photon Source, Argonne National Laboratory, Lemont, IL 60439, USA; Paul Scherrer Institut, Switzerland

**Keywords:** hybrid machine-learning segmentation, X-ray micro-computed tomography, image quantitative analysis, gas hydrates

## Abstract

Fast multi-phase processes in methane hydrate bearing samples pose a challenge for quantitative micro-computed tomography study and experiment steering due to complex tomographic data analysis involving time-consuming segmentation procedures. This is because of the sample’s multi-scale structure, which changes over time, low contrast between solid and fluid materials, and the large amount of data acquired during dynamic processes. Here, a hybrid approach is proposed for the automatic segmentation of tomographic data from time-resolved imaging of methane gas-hydrate formation in sandy granular media, which includes a deep-learning 3D U-Net model. To prepare a training dataset for the 3D U-Net, a technique to automate data labeling based on sample-specific information about the mineral matrix immobility and occasional fluid movement in pores is proposed. Automatic segmentation allowed for studying properties of the hydrate growth in pores, as well as dynamic processes such as incremental flow and redistribution of pore brine. Results of the quantitative analysis showed that for typical gas-hydrate stability parameters (100 bar methane pressure, 7°C temperature) the rate of formation is slow (less than 1% per hour), after which the surface area of contact between brine and gas increases, resulting in faster formation (2.5% per hour). Hydrate growth reaches the saturation point after 11 h of the experiment. Finally, the efficacy of the proposed segmentation scheme in on-the-fly automatic data analysis and experiment steering with zooming to regions of interest is demonstrated.

## Abstract 2

A new hybrid machine-learning approach for the automatic segmentation of dynamic computed tomography images during methane hydrate formation in sandy samples is presented. The algorithm allows for accurate and fast segmentation of gas hydrate changes and fluid flow in the low-contrast environment that is the main step to perform automatic quantitative analysis of processes in hydrate-bearing samples.

## 1. Introduction

*In situ* X-ray computed tomography (CT) imaging is crucial for studying geomaterials, as it enables the observation of thermo-baric conditions similar to those found in the Earth’s interior (Fusseis *et al.*, 2014 ▸). Synchrotron radiation sources are particularly useful for time-resolved CT imaging of fast processes in geomaterials, such as geomechanical deformation and rock failure (Zhang *et al.*, 2022 ▸), gas-hydrate formation and dissociation in porous samples (Nikitin *et al.*, 2020 ▸, 2021 ▸), and dynamic fluid flow through pore pathways (Dobson *et al.*, 2016 ▸). These techniques provide valuable insights into the pore-scale processes and their effect on the macroscopic properties of geomaterials.

Accurate segmentation of 3D volumes is crucial in synchrotron imaging, particularly when dealing with fast data acquisition rates. However, low data contrast and artifacts in reconstructions can make automatic segmentation challenging, leading users to resort to slow manual segmentation. This can be observed, for instance, in materials science and geology: low-contrast phases observed in CT imaging of carbonate samples (Alqahtani *et al.*, 2022 ▸) and carbon/epoxy woven composites (Sinchuk *et al.*, 2020 ▸). Other examples include nano-CT in battery research (Hallot *et al.*, 2022 ▸) or chemical science (Kim *et al.*, 2023 ▸). Fast segmentation procedures can aid in localizing regions of interest and selecting time intervals for studying dynamic phenomena, as well as providing quantitative estimations of sample components for input into digital rock physics simulations (Wang *et al.*, 2015 ▸; Sell *et al.*, 2016 ▸). The accuracy of segmentation results can significantly impact image-computed rock properties, potentially affecting the accuracy of numerical simulations (Saxena *et al.*, 2017 ▸; Rezaei *et al.*, 2019 ▸).

Micro-CT images can be segmented using various methods, including global thresholding (Iassonov *et al.*, 2009 ▸), marker-based watershed algorithms (Zhang & Duanquan, 2012 ▸) and clustering algorithms (Kang *et al.*, 2009 ▸). However, the segmentation of multiphase materials with low contrast, particularly in the context of rock samples, presents limitations for these conventional methods. Therefore, better segmentation techniques are needed for the automatic analysis of images with low-contrast materials.

The global thresholding method, in the case of images with close gray-level averages, can be improved by the Gaussian mixture model (GMM) for separating materials (Huang & Chau, 2008 ▸). Another approach is to use deep artificial neural networks for image processing and segmentation (Egmont-Petersen *et al.*, 2002 ▸). Deep convolutional neural networks (CNNs) are actively developing due to the increase in computational performance of graphical processing units (GPUs). In segmenting scanning electron microscope images, they produce results of superior quality compared with conventional algorithms (Ciresan *et al.*, 2012 ▸). Further segmentation improvements were demonstrated with the architecture of fully convolutional neural networks (FCNNs) (Long *et al.*, 2015 ▸). One of the most popular FCNN architectures is the well known U-Net (Ronneberger *et al.*, 2015 ▸), which was also extended to 3D (Çiçek *et al.*, 2016 ▸). Unlike previous FCNN models, the U-Net architecture allows training on a small dataset and is widely used for solving quantification tasks in various scientific studies (Falk *et al.*, 2019 ▸). However, despite the advancements in neural network models and the availability of open packages, their use for segmenting CT images remains limited. This is mostly because of the difficulty in preparing the training datasets, particularly for the 3D case.

In this paper, we develop a new method for automatic segmentation of dynamic CT images for the experiments in which the multi-phase flow takes place in a sample pore space. First, we suggest a method for forming a marked training set in the case of low contrast between the solid and liquid phases. The rock matrix is immobile throughout the experiment while the pore fluid moves around occasionally. Thus it is possible to find regions for which there exist two scans: with pore-space filled with gas and with liquid. In the case of gas in pores one can easily segment the matrix using a threshold algorithm. This segmentation and another scan with fluid-filled pores can be used in the training set. Given the training set we suggest the segmentation strategy that consists of two sequential steps: (1) training and applying a 3D U-Net neural network to segment the rock matrix for the whole sample; and (2) statistical clustering of the pore-space phases using the GMM. The method employs the 3D U-Net to effectively distinguish the low-contrast rock matrix from brine-containing pore phases. The resulting matrix masks can be used for all time steps of the experiment (assuming rock matrix immobility). In addition to segmenting the mineral matrix, our method employs an unsupervised clustering algorithm to automatically adjust the threshold for separating the remaining phases with sufficient contrast. This enables the segmentation of materials with complex geometries, mixed compositions and variable density, eliminating the need to create a training set specifically adapted to these challenging cases.

The effectiveness of the proposed method was demonstrated through an automatic quantitative analysis of CT data from dynamic imaging of methane hydrate formation in sandy samples (Nikitin *et al.*, 2020 ▸). Furthermore, we showed the applicability of the trained model in on-the-fly experiment steering, particularly in scenarios where information about future time steps is unavailable and segmentation needs to be performed in real time. We conducted on-the-fly data segmentation during continuous scanning of the sample in low resolution, using the segmentation results to identify regions of interest for subsequent high-resolution scanning. Note that the low-contrast issue in hydrate-bearing media is not limited to the components used in this article. It is also present in other samples, such as hydrate-bearing coal samples (Nikitin *et al.*, 2021 ▸) and sand samples with NaBr and KI solutions with different concentrations (Chen *et al.*, 2020 ▸).

The paper is organized as follows. In Section 2 we briefly describe the experimental setup, data acquisition and data processing procedures used to generate 3D volumes for further segmentation. In Section 3 we introduce our two-step automatic segmentation approach involving the usage of U-Net architectures (2D and 3D) and the GMM. Section 4.1 presents the applications of the proposed technique for quantitative analysis of the segmentation results and discusses new insights about the formation process. The experiment steering with on-the-fly segmentation is demonstrated in Section 4.2. Conclusions and outlook for further studies are given in Section 5.

## 2. Data description and low-contrast problem definition

The tomographic data used to develop our segmentation technique were acquired during a dynamic *in situ* experiment at the bending magnet beamline 2-BM of the Advanced Photon Source, Argonne National Laboratory [see Nikitin *et al.* (2020 ▸) for details]. For gas-hydrate formation, we filled an environmental cell with silica sand and water, supplied it with methane gas under high pressure, and continuously cooled it at −7°C. The cell was rotated, and several subvolumes of the sample were scanned with a parallel X-ray beam every 15 minutes to capture the sample states without gas hydrates and the states where the hydrate is continuously forming by filling the pore space. Acquisition of one tomographic dataset corresponding to a 180° sample rotation took 70 s.

To obtain qualitative tomographic reconstructions, we used the *TomocuPy* package (Nikitin, 2023 ▸) to organize a reconstruction pipeline. The reconstruction pipeline included common processing functions such as ring removal, phase retrieval filtering, and filtered backprojection implemented via the log-polar-based method (Andersson *et al.*, 2016 ▸).

The dynamic synchrotron experiment on gas-hydrate formation generated more than 200 GB of reconstructed 3D images per experimental day. Each reconstructed volume was in 32-bit precision and had a size of 1224 × 1224 × 512, corresponding to a real sample volume of 4.3 mm × 4.3 mm × 1.8 mm with a voxel size of 3.45 µm × 3.45 µm × 3.45 µm. Each 3D image was reconstructed from 1500 8-bit projections of size 2448 × 1024. The reconstruction process for one dataset took approximately 40 s using an Nvidia Tesla V100 GPU. For the development and testing of our segmentation algorithm, we used 66 3D tomographic images at various times during the hydrate formation process.

According to the presence and quantity of each phase (sand grains, methane hydrate, NaBr brine and methane gas) during the formation process, we have distinguished three main experimental stages: (1) before the methane hydrate formation, (2) during the methane hydrate formation, and (3) after the methane hydrate formation. In the first stage, tomographic images contain methane gas, sand grains and NaBr brine. The second stage covers the sample states with methane gas, sand grains, methane hydrate, NaBr brine and a mixture of NaBr brine and methane hydrate. In the third stage the hydrate formation mostly stops. Rare localized bodies of brine-saturated phases still exist but one can easily find regions free of brine-saturated phases by visual inspection.

**Figure 1:**
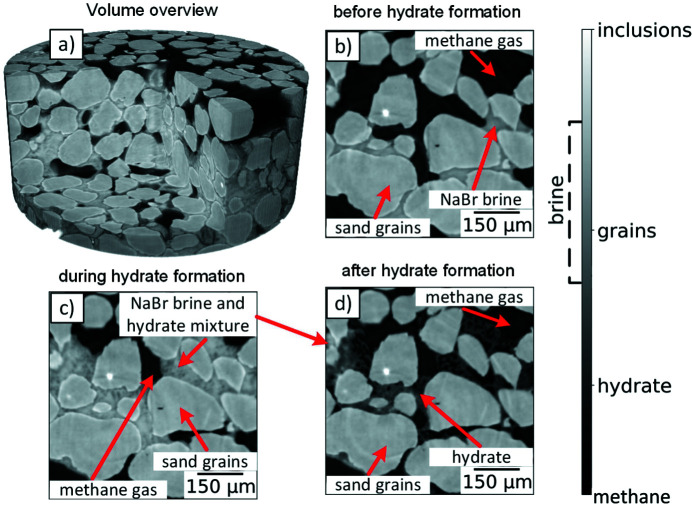
An example of 3D reconstruction of the hydrate containing sandy sample (*a*). Cropped parts of the slices show a typical content of tomographic data obtained at the main stages of the tomographic experiment (*b*, *c*, *d*).

An example of a reconstructed volume after a cylindrical cut is shown in Fig. 1 ▸(*a*). Panels (*b*), (*c*) and (*d*) of the same figure show three examples of cropped slices obtained at the different experimental stages. The figure is equipped with notes indicating different materials: black color corresponds to the methane gas, dark gray to the gas hydrate, light gray to salty water (NaBr brine) and lightest gray to sand grains. One can also observe regions with a mixture of the NaBr brine and the hydrate.

Micro-computed tomography imaging of methane-hydrate-bearing samples poses challenges due to the similar densities of water and methane hydrate. To address this issue, the use of salt brine as a phase contrast agent is a common practice. Salt brine helps to separate the hydrate and water in images by increasing the contrast between phases. The use of salt brine in this way is also reflective of the natural conditions under which hydrates form in the bottom sediments of the sea, where the water has some salinity. Lei & Santamarina (2018 ▸) demonstrate efficient phase-contrast-enhancing methods using NaBr and KI brines as hydrate-forming fluids, as opposed to regular formation with deionized water.

In this work, we tested different levels of brine salinity and chose 10% NaBr as it demonstrated a more favorable hydrate–water contrast for further segmentation procedures. However, increasing the contrast between one pair of phases reduces the contrast between other pairs of phases. Specifically, the phase contrast between brine and sand grains is significantly reduced with the appearance of brine. Conventional segmentation algorithms based on the gray-level separation become ineffective in this case.

**Figure 2:**
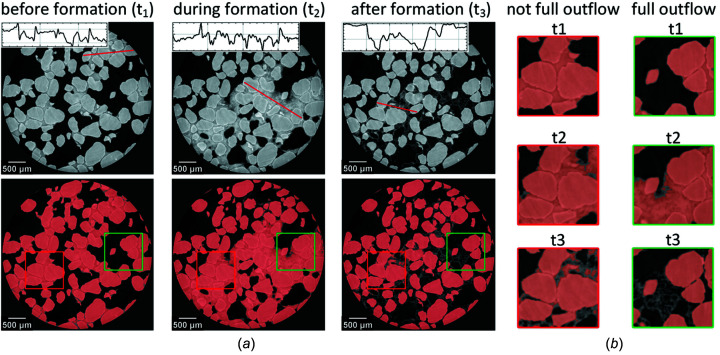
Failed segmentation of sand grains using a threshold method due to gray-level instability within the grains and low contrast between the grain phase and brine-saturated phases within the pore space (*a*). Demonstration of selected sub-regions of the slices depicting complete brine outflow (green rectangle) and regions with remaining brine-saturated phases (red rectangle) after hydrate formation (*b*).

Figure 2 ▸ demonstrates an example of incorrect segmentation based on the gray-level separation. Each slice in the top row of Fig. 2 ▸(*a*) includes a panel that displays the profile along the red line, highlighting the instability of gray levels within the grains, as well as the similarity in gray levels between the grains and NaBr saturated phases. The bottom row of Fig. 2 ▸(*a*) shows the results of applying the threshold algorithm for grain segmentation. In Fig. 2 ▸(*b*), specific areas are highlighted in the segmented images from each stage of the experiment. A green rectangle represents an example of the sample area where the entire brine flows out during the hydrate formation process. In these areas, the threshold algorithm can effectively segment the grains after hydrate formation. Conversely, a red rectangle indicates an area where the brine remains static throughout the experiment, making the threshold algorithm unsuitable for segmentation.

In the following we will describe a segmentation approach utilizing the regions of the sample where the brine outflow occurs after hydrate formation. This approach will facilitate the creation of masks for the grains using the threshold algorithm.

## 3. Segmentation procedure

In this section we present basic concepts and implementation details of the two-step segmentation technique developed for segmenting dynamic CT data obtained during the methane hydrate formation in sandy samples. The proposed segmentation procedure involves application of the following methods:

(i) Sand grains segmentation using the U-Net model.

(ii) Pore space phases segmentation using a clustering algorithm based on the GMM.

The first step is needed to separate sand grains and brine filled phases (NaBr brine and the mixture of NaBr brine and methane hydrate). For this, we apply semantic segmentation of grains by using models based on deep fully convolutional U-Net networks (Ronneberger *et al.*, 2015 ▸). The approach is well applicable for grains segmentation as they have distinct shapes and sizes. Moreover, in Section 3.1.1 we describe an approach for automatic labeling for training dataset preparation for both 3D and 2D models, which is important for using supervised models. At the second step, the segmented areas of sand grains are subtracted from the images, and separation of the remaining phases is carried out with the clustering algorithm based on the GMM (Huang & Chau, 2008 ▸).

The whole segmentation procedure is implemented in Python using the *TensorFlow* and *scikit-learn* packages. We have made our segmentation approach publicly available on GitHub (https://github.com/mikhail-qwerty/Unet-GMM_segmentation). The code implements a segmentation workflow on both individual tomographic volumes and a series of sequential tomographic volumes. The U-Net segmentation requires training a new model or loading already trained weights. The results of grains segmentation are used as a mask for further clustering of the remaining phases. The main input parameters for the clustering model include the number of clusters, average intensity values for the clusters, and options for splitting data into parts for memory optimization. It is also possible to configure other clustering parameters based on the *scikit-learn* package documentation. These settings enable the setup of the initial model for fitting a mixed distribution model. The procedure is executed only for the first time step in the case of time series clustering. Once the model has been fitted, the distribution parameters are saved and used as input for the next time step. Consequently, after training the U-Net and selecting the initial parameters for the GMM model, segmentation does not necessitate user intervention and functions automatically.

### 3.1. U-Net based models for sand grains segmentation

For the grains segmentation we used both 2D and 3D U-Net implementations based on the models described by Ronneberger *et al.* (2015 ▸) and Çiçek *et al.* (2016 ▸). The architecture of the U-Net models can be divided into the encoder and the decoder parts. The encoder consists of the convolutional and downsampling layers applied step by step to compute feature maps of the input data. The decoder takes low-resolution feature representations and generates the mask using the transposed convolutional and convolutional layers. The resulting masks have the same size as the input data.

**Figure 3:**
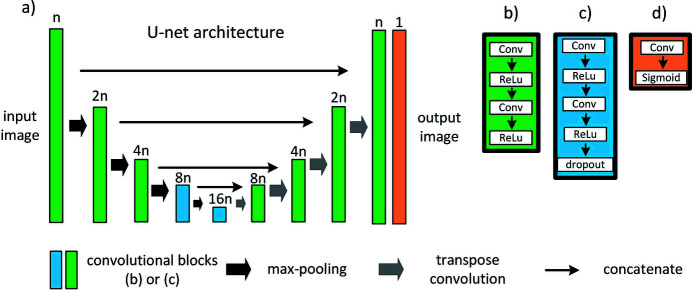
Schematic overview of the 2D and 3D U-Net architectures used in this work. Colored squares denote the convolutional blocks and the arrows denote different math operations.

A detailed description of the proposed U-Net architecture is presented in Fig. 3 ▸. One can see that the encoder and the decoder parts consist of four convolutional blocks [Fig. 3 ▸(*a*)]. Each block is highlighted by the color depending on the layers it contains. So, we used an architecture with two types of convolutional blocks and one final convolutional layer. Green color indicates the blocks consisting of two sequential convolutional layers with the *ReLu* activation function [Fig. 3 ▸(*b*)]. Blue color indicates the blocks with two convolutional layers followed by dropout [Fig. 3 ▸(*c*)]. Orange color indicates the final convolutional layer with a kernel size of 1 pixel and the sigmoid activation function [Fig. 3 ▸(*d*)].

We implemented the described architecture in both 2D and 3D. The number of filters for the first convolutional block was 32 and 16 for the 2D and 3D models, respectively.

#### 3.1.1. Preparation of training and validation datasets

The supervised learning strategy we propose requires labeled datasets for training. Manual labeling of tomographic images is time-consuming, especially in 3D; therefore, in this work we avoided manual data labeling following the strategy described below.

To automate the data labeling procedure, we utilize one of the main properties of hydrate formation in porous media: sand grains remain motionless (unlike a freezing process), while pore brine is mobile and can disappear by the end of the experiment due to conversion into gas hydrate or outflow from the field of view to another hydrate growth region, as described in detail by Nikitin *et al.* (2020 ▸). As mentioned earlier, CT images at the end of the hydrate formation exhibit regions with an absence of brine-saturated phases. These regions consist of methane gas, methane gas hydrate, sand grains and constitute only a small portion of the overall hydrate-containing sample. These areas are primarily located in regions far from the initial regions of the formation process. Thus, the first step in preparing the training dataset is to identify such regions.

**Figure 4:**
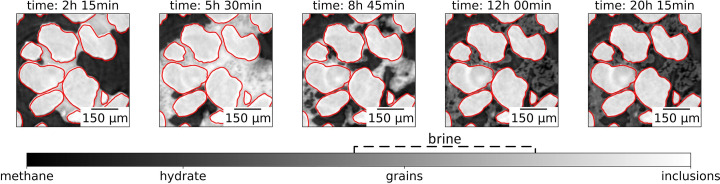
Demonstration that grains are motionless during the hydrate formation process. The red curves indicate that the borders of the grains at the beginning of the experiment have the same positions in all images.

Given that the phases present in these regions are well separated in the gray-level images, conventional thresholding algorithms can be used to automatically segment the grains, as demonstrated in the right-hand panel of Fig. 4 ▸. Because the grains remain motionless throughout the experiment, the resulting grain masks can be applied to images from previous time steps. Thus, we need only select regions and times when brine was present in the pore space, as illustrated in the first two panels of Fig. 4 ▸.

Following this automated labeling approach we prepared a labeled dataset consisting of 1134 sub-volumes (256 × 256 × 256) overlapping by not more than 145 pixels. 80% of this dataset was used for training and validation. The remaining 20% was used for testing. The training dataset for 2D segmentation was prepared by slicing the 3D sub-volumes.

#### 3.1.2. Training results and quality comparison

**Figure 5:**
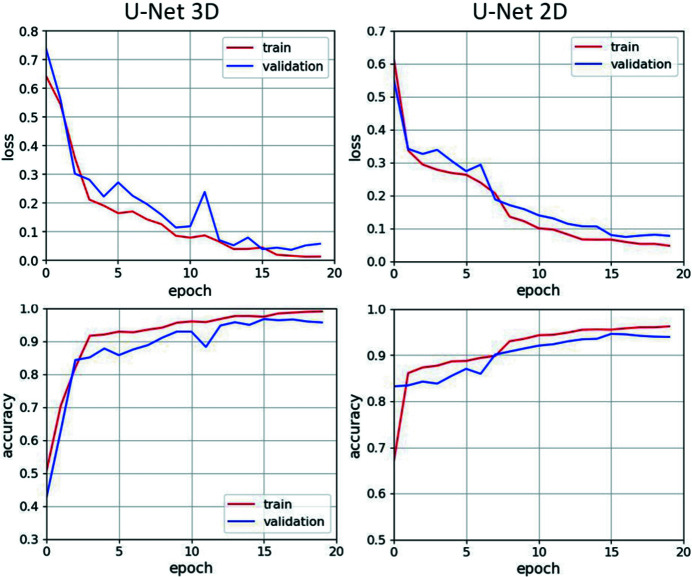
Learning curves of the U-Net 3D (left column) and U-Net 2D (right column) models. The curves display the accuracy and loss function measurements for both the train and validation datasets during training.

The 2D and 3D segmentation models were trained using the same deep-learning training strategy. For explanation of all machine-learning terms and techniques used in this section we refer to Goodfellow *et al.* (2016 ▸). The training procedure involved the *binary cross-entropy* loss function minimization with the *Adam* optimizer (learning rate is 10^−4^, metric is *accuracy*). The metric and loss function values on the training and validation sets were monitored during training to avoid the model overfitting and determine the optimal number of training epochs (Fig. 5 ▸). As a result, we chose the number of epochs to be equal to 15. Segmentation quality on the test dataset was measured by the *mIoU* metric. To analyze the performance of 2D and 3D U-Nets for processing large datasets we measured the computational time for the training and prediction stages.

Table 1 ▸ presents the segmentation quality and performance results, and the training time (for 15 epochs) was estimated by averaging ten independent training runs on the same dataset. Prediction time was calculated for a 1224 × 1224 × 512 image volume divided into 75 patches of size 256 × 256 × 256 with overlapping by 14 pixels. The training and testing procedures were implemented in Python using the *TensorFlow* (Version 2.7.0) package, and the performance testing was carried out on an NVidia Tesla V100 GPU with 16 GB of memory. Data for both models were transmitted sequentially. Note that the 3D model was faster by 5 s than the 2D model in prediction, but the 2D model was 20 times faster in training. The *mIoU* metric demonstrated that the 3D model outperformed the 2D model by 4% in terms of segmentation quality.

**Figure 6:**
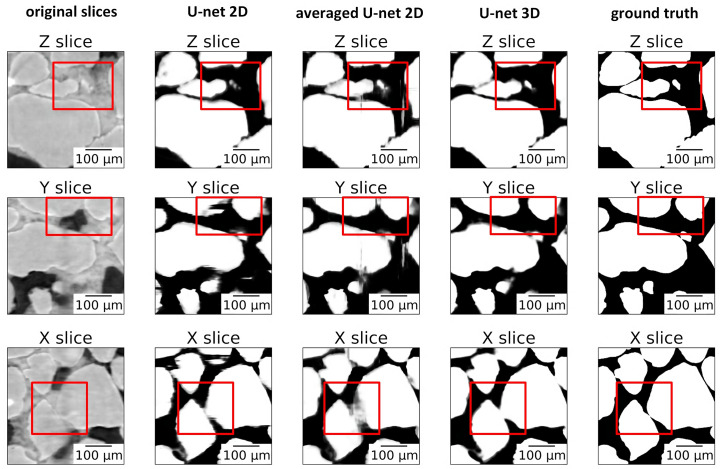
Comparison of segmentation results by the U-Net 2D, averaged U-Net 2D and U-Net 3D neural networks applied to a sub-volume from the test sample. Each panel shows *X*, *Y* and *Z* central slices from the sub-volume. Red rectangles correspond to the regions with significant differences in segmentation quality.

Figure 6 ▸ demonstrates a comparison of the grains segmentation quality for the 3D and 2D U-Net models. Each row of the figure shows the central slice of a sub-volume from the test dataset: horizontal slice in the upper row (labeled *Z*-slice), vertical slice orthogonal to the *Y*-axis in the middle row (labeled *Y*-slice), vertical slice orthogonal to the *X*-axis in the bottom row (labeled *X*-slice). One can see the original slices in the leftmost column and the labeled slices (ground truth) in the rightmost column. The three columns in the middle show segmentation results of applying different U-Net models: 2D U-Net applied to horizontal slices in a slice-by-slice manner (second column); 2D U-Nets applied in a slice-by-slice manner in directions orthogonal to axes *X*, *Y* and *Z*, respectively, followed by averaging the results (third column); 3D U-Net applied to the whole volume (fourth column).

Note that 2D segmentation (second and third columns in Fig. 6 ▸) results in ‘comb’-type artifacts caused by the fact that the 2D model does not take into account information from neighboring slices, and may easily build non-smooth boundaries in the direction of the slice sliding. The areas with the strongest artifacts are highlighted by the red squares. On the contrary, the 3D model is able to build smooth boundaries in all directions and provides the best segmentation result. Our conclusions are in line with the assumption of Deniz *et al.* (2018 ▸) that the end-to-end 3D CNN segmentation model is more favorable for segmenting challenging 3D images.

### 3.2. Clustering with the GMM

After extracting segmented sand grains from the original image, the remaining phases (gas, hydrate, brine and hydrate mixture, brine) were separated by the global threshold method. Determining the global threshold is challenging because of unstable gray-level intervals associated with changing NaBr brine concentration. These instabilities were mainly due to the effect of salt ions exclusion from the water consumed for the hydrate formation process (Chen *et al.*, 2020 ▸). Thus the hydrate formation slowly increases the brine salinity in time, shifting the gray-level distribution between phases. As a result, we need to determine the gray-level thresholding ranges at each time step.

In this work, we used a clustering algorithm based on the GMM to automatically determine the global threshold at each time step. This model allows the mixed normal distribution to be decomposed into the sum of Gaussians, followed by iteratively calculating their parameters with the Expectation Maximization algorithm (Balafar, 2014 ▸). Definition of the starting GMM requires setting the number of mixture components and initializing parameters for Gaussian distributions. We defined the number of components equal to four according to the maximum number of phases that can be presented in our CT data. To include the time dependence of the data, we used the means, covariances and weights calculated at the previous time step as parameters for initializing the starting model at the current time step. For the first time step, the model was initialized using the *k*-means algorithm or by manual definition of the parameters.

**Figure 7:**
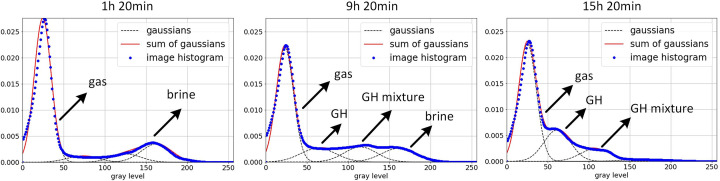
Histogram approximation by the GMM. Each graph corresponds to CT data associated with different times in the hydrate formation experiment. The data histogram is shown by blue dots, Gaussians fitted with GMM by black dots, and sum of the Gaussians by a red line.

Figure 7 ▸ shows the results of the mixture distribution decomposition using the approach described above. Three time steps were chosen as examples describing the main stages of the experiment: before hydrate formation (1 h 20 min), during hydrate formation (9 h 20 min) and after hydrate formation (15 h 20 min). The experiment time is measured from the moment when pressure and temperature conditions for the hydrate formation became stable. Each panel in Fig. 7 ▸ includes experimental and predicted probability density functions (PDFs) marked by blue and red colors, respectively. Gaussian distributions for each phase from the GMM model are marked by the black dashed line. The experimental PDF is calculated from the histogram of the images, while the predicted PDF is calculated as a sum of predicted Gaussian distributions. Black arrows indicate phases corresponding to each Gaussian. One can see that the proposed mixed distribution decomposition into four components gives a good agreement between the experimental and predicted PDF. These four components were associated with the following phases: sand grains, methane gas (gas), methane gas hydrate (GH), methane hydrate and NaBr brine mixture (GH mixture), NaBr mixture.

**Figure 8:**
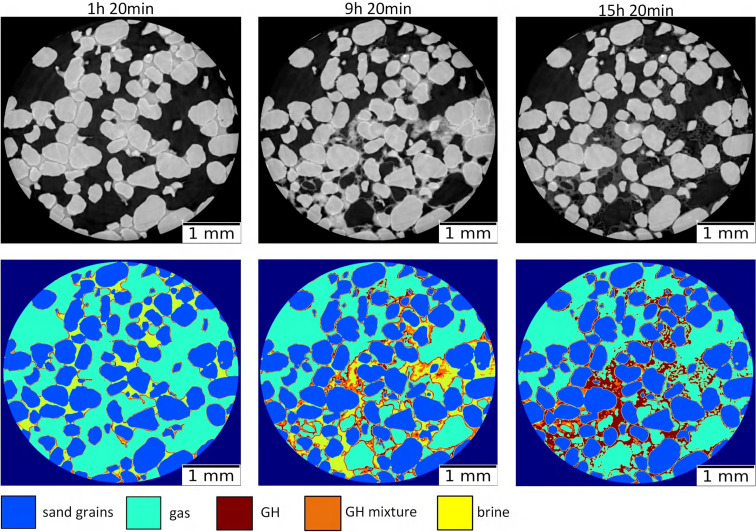
Extracted horizontal slices from the clustered 3D sample volumes by the proposed two-step segmentation model. Each panel corresponds to CT data from different experiment times.

Figure 8 ▸ shows the results of clustering CT images with the proposed two-step segmentation algorithm. The top row shows central slices of 3D image sample volumes at different experiment times. Histogram approximation plots in Fig. 7 ▸ were prepared by making use of these data. After decomposing the gray-level curve into separate Gaussians one can use thresholding for separating corresponding phases. Thresholding boundaries were chosen as intersections between the Gaussians and yielded segmentation masks shown in the bottom row of Fig. 8 ▸. Colors correspond to different phases in the GMM: sand grains (blue), gas (green), gas hydrate (brown), mixture of brine and hydrate (orange) and pure brine (yellow).

## 4. Results and discussion

The proposed technique is demonstrated in the following subsections for two applications: (1) quantitative estimation of phases during the gas-hydrate formation process, and (2) estimation of regions with dynamic events during hydrate formation for automated focusing and scanning with high resolution.

### 4.1. Quantification of phases during dynamic imaging

**Figure 9:**
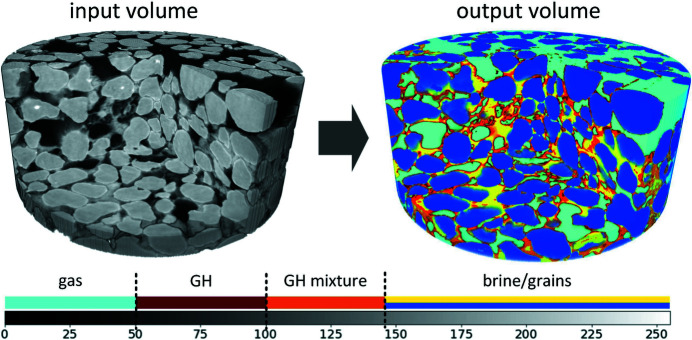
Example of applying the proposed segmentation method in 3D (color code is the same as in Fig. 8 ▸).

Let us discuss some applications of the proposed segmentation method. First, we can use the developed two-step segmentation algorithm to process 3D CT image volumes in an automatic manner, where each CT volume is provided as input and its automatic segmentation is formed as output, see Fig. 9 ▸. The colors of the materials here are the same as in Fig. 8 ▸: sand grains (blue), gas (green), gas hydrate (brown), pure brine (yellow), mixture of brine and gas hydrate (orange). Note that the solid sample matrix consists of two materials: sand grains (invariant) and gas hydrate (changing in time). Thus, as the first application of our segmentation we can consider the monitoring of the 3D pore space geometry changing in time. The resulting time-resolved 3D models can be further used in digital rock physics simulations for estimating changes in the sample permeability and in other petrophysical properties during the hydrate formation.

Another application is related to the quantitative estimation of the sample material changes in time. Pore-space saturation of each particular phase can be computed as a sum of all voxels corresponding to this phase, divided by the sum of voxels corresponding to the entire pore space. For example, methane gas saturation in pores of the volume in Fig. 1 ▸ is 61.8%. The situation is more complicated for such materials as the pore brine or gas hydrate. Based on our GMM, the gas hydrate is presented in two phases: as the GH phase (brown color in Fig. 8 ▸) and as a part of the GH mixture (orange color in Fig. 8 ▸). In the latter case, voxels should be decomposed into relative amounts of brine and hydrate. Here we assume a linear dependence of the relative content of these phases on the gray level within the interval of the GH mixture phase. Therefore, for quantitative estimation of the gas-hydrate material in a 3D volume we compute the fraction of the GH-mixture voxels following our linear dependence assumption and add it to the number of GH phase voxels. Similar estimations can be done for the pore brine material. To compute the gas hydrate and brine saturation, the obtained values are divided by the number of voxels representing the whole pore-space volume.

**Figure 10:**
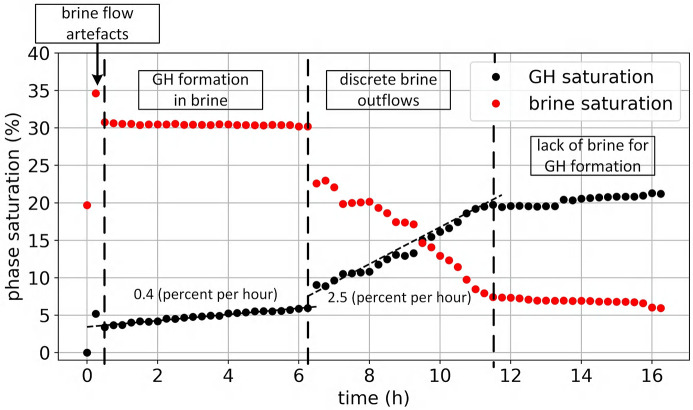
Hydrate and water saturation plots over the whole hydrate growth process. The red curve corresponds to water saturation, the black curve to hydrate saturation.

In Fig. 10 ▸ we show the saturation of the brine (red curve) and hydrate (black curve) at different times during the experiment. These automatically generated curves give new insights into the hydrate formation process. First, we see two distinct periods of the hydrate growth. Gas-hydrate growth is slow during the first 6 h. Close examination of the images revealed that the hydrate growth rate changed after a massive volume of brine moved away out of the scanned region shortly after 6 h. Apparently, the gas transfer into massive water volumes is slow resulting in a slower hydrate-formation rate of 0.4% per hour – see the interval 0–6 h in Fig. 10 ▸. After massive water outflow the surface of the brine–gas contact increases resulting in a faster gas-hydrate formation rate of 2.5% per hour – see the interval 6.5–11 h in Fig. 10 ▸. Finally, the hydrate growth reaches saturation after 11 h of the experiment.

### 4.2. Application to the dynamic imaging experiment with steering

In this section we describe the application of the developed technique for real-time segmentation of data from a multi-resolution dynamic experiment with steering. In the experiment, we utilize segmentation results to identify pores exhibiting dynamic events, such as brine outflows with the initiation of the hydrate growth. These regions are then scanned at higher resolutions for more detailed imaging of the methane hydrate formation process.

As a sample for the experiment we used silica sand filled with a 10% NaBr brine (10 wt%) for the contrast enchantment. The entire sample was represented as a cylinder measuring 2 cm in height and 0.5 cm in diameter. Low-resolution scanning was carried out using a 1.1× optical lens, providing a pixel size of 3.45 µm, while high-resolution scanning was performed using a 5× lens, providing a pixel size of 0.69 µm.

The goal of steering was to monitor the gas-hydrate formation process at low spatial resolution (1.1× lens) and then focus into regions where fast water outflows have occurred spontaneously. While scanning the sample with low resolution we applied our segmentation workflow on-the-fly with already trained U-Net and formed GMMs constructed by using data from the experiment described in previous sections. Based on the segmentation results, we selected pores with significant fluid flows and highest amount of the hydrate, and then automatically zoomed in to these pore regions for higher-resolution (5× lens) scanning. Such steering allowed us to capture the initiation of the hydrate formation process and monitor the evolution of the process in high resolution. Note that without the proposed on-the-fly segmentation we would be able to detect water flows with a regular thresholding method; however, we would not be able to check whether the pore contains the hydrate or not. The proposed method prevented selecting regions of interest where the hydrate is not supposed to form, and, as a result, prevented conducting the whole experiment several times.

**Figure 11:**
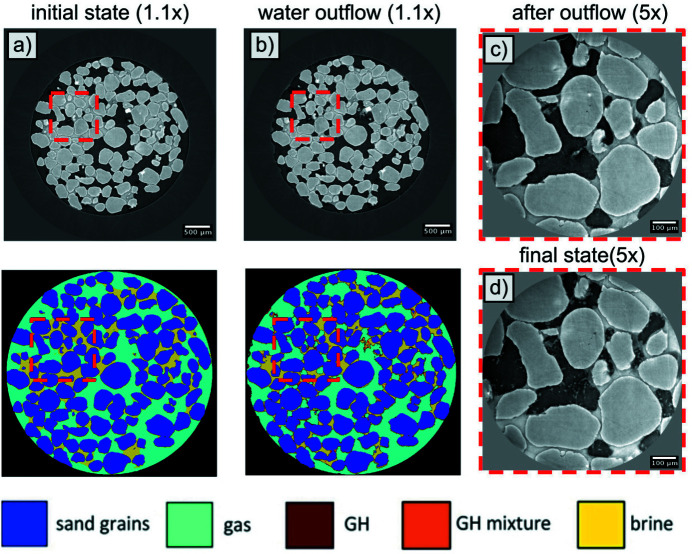
Horizontal slices of reconstructed volumes at low resolution before and after water redistribution are shown in (*a*) and (*b*), respectively. The corresponding segmentation results are shown below them. Panels (*c*) and (*d*) correspond to the selected ROI scanned with the 5× lens after the water outflow and after the hydrate formation, respectively.

Figs. 11 ▸(*a*) and 11(*b*) show examples of slices through reconstructed volumes at low spatial resolution for the sample state before and after brine outflow. The segmentation results are shown below them. The region of interest with water outflow is marked by a red dashed rectangle. After the low-resolution scan, this region was imaged with the 5× lens [Fig. 11 ▸(*c*)], where the gas-hydrate structure begins to form on the water–gas interface. The region was further continuously scanned until the final state [Fig. 11 ▸(*d*)] was reached.

As a result, our trained segmentation model enhanced the information content of CT images during the dynamic process of hydrate formation. Using the segmentation results, we were able to identify and select regions of interest containing the target phase (methane gas hydrate) and the target event (fluid outflow). Furthermore, this valuable information was obtained in real time, which is crucial for dynamic imaging of processes and on-the-fly experiment steering.

## 5. Conclusions

With the development of new powerful synchrotron sources, data acquisition rates will become significantly higher, making it possible for X-ray beamline instruments to generate 100× more data per experiment (Chenevier & Joly, 2018 ▸; Fornek, 2019 ▸). Large data volumes require automatic segmentation techniques, which is particularly challenging for imaging gas-hydrate formation in granular samples. The phases have weak gray-level contrast (grains and brine) that is also evolving in time (brine salinity increases during the hydrate formation).

We proposed a two-step segmentation workflow. Firstly, we utilized a 3D U-Net architecture to tackle the challenging task of separating sand grains from brine-saturated phases. To train the model, we developed an automated workflow that is based on the mineral matrix immobility (common property of the hydrate formation process). This allowed the use of a 3D U-Net, and we have shown that this helps to eliminate artifacts in images compared with a 2D U-Net slice-by-slice and averaged approaches. Next, we segmented the remaining phases using the GMM to adapt the global threshold levels that vary with time. However, a limitation for the application of phase separation by the GMM model is the requirement for sufficient phase contrast between methane hydrate and water, which can be achieved by using heavy salt brines such as NaBr or KI.

We showed application of the proposed hybrid machine-learning method for quantitative estimation of the hydrate-saturation changes in time and for brine outflows detection while dynamic experiment with steering. The hydrate-saturation curve revealed two distinct periods of the hydrate growth: slow hydrate growth in the beginning, followed by a five-times faster hydrate growth after the massive water outflow. These observations are important for a better understanding of the kinetics of the hydrate formation in porous medium – massive water outflows in pores improve brine–gas contact facilitating the hydrate formation.

The proposed method may become an essential tool for imaging and analyzing gas hydrates in high temporal and spatial resolution. In fast-evolving dynamic systems, automatic segmentation, classification and detection may allow for steering tomographic experiments, *e.g.* changing environmental conditions (pressure, temperature, electrical charge) based on real-time sample states. Artificial-intelligence-based steering techniques will play a very important role in future complex dynamic experiments at brilliant light sources.

We believe that our automated data labeling approach may benefit researchers facing similar segmentation challenges and working with similar experimental processes, such as melting/freezing permafrost samples, heating up coal media with diverse inclusions, *etc*. 

## Figures and Tables

**Table 1:** Quality and performance testing of 2D and 3D U-Net models for segmenting sand grains in 1224 × 1224 × 512 reconstructions of hydrate-bearing samples

Neural network architecture	Prediction time (one volume)	Training time (15 epochs)	*mIoU*
2D U-Net	19 s	5.4 min	0.931
3D U-Net	14 s	102 min	0.976

## References

[bb1] Alqahtani, N. J., Niu, Y., Wang, Y. D., Chung, T., Lanetc, Z., Zhuravljov, A., Armstrong, R. T. & Mostaghimi, P. (2022). *Transport Porous Media*, **143**, 497–525.

[bb2] Andersson, F., Carlsson, M. & Nikitin, V. V. (2016). *SIAM J. Imaging Sci.* **9**, 637–664.

[bb3] Balafar, M. (2014). *Artif. Intell. Rev.* **41**, 429–439.

[bb4] Chen, X., Espinoza, D. N., Luo, J. S., Tisato, N. & Flemings, P. B. (2020). *Mar. Petrol. Geol.* **117**, 104340.

[bb5] Chenevier, D. & Joly, A. (2018). *Synchrotron Radiat. News*, **31**(1), 32–35.

[bb6] Çiçek, Ö., Abdulkadir, A., Lienkamp, S. S., Brox, T. & Ronneberger, O. (2016). *International Conference on Medical Image Computing and Computer-Assisted Intervention (MICCAI 2016)*, 17–21 October 2016, Athens, Greece, pp. 424–432. Springer.

[bb7] Ciresan, D., Giusti, A., Gambardella, L. & Schmidhuber, J. (2012). *Adv. Neural Inf. Process. Syst.* **25**, 2843–2851.

[bb8] Deniz, C. M., Xiang, S., Hallyburton, R. S., Welbeck, A., Babb, J. S., Honig, S., Cho, K. & Chang, G. (2018). *Sci. Rep.* **8**, 16485. 10.1038/s41598-018-34817-6 PMC6220200 30405145

[bb9] Dobson, K. J., Coban, S. B., McDonald, S. A., Walsh, J. N., Atwood, R. C. & Withers, P. J. (2016). *Solid Earth*, **7**, 1059–1073.

[bb10] Egmont-Petersen, M., de Ridder, D. & Handels, H. (2002). *Pattern Recognit.* **35**, 2279–2301.

[bb11] Falk, T., Mai, D., Bensch, R., Çiçek, Ö., Abdulkadir, A., Marrakchi, Y., Böhm, A., Deubner, J., Jäckel, Z., Seiwald, K., Dovzhenko, A., Tietz, O., Dal Bosco, C., Walsh, S., Saltukoglu, D., Tay, T. L., Prinz, M., Palme, K., Simons, M., Diester, I., Brox, T. & Ronneberger, O. (2019). *Nat. Methods*, **16**, 67–70. 10.1038/s41592-018-0261-2 30559429

[bb12] Fornek, T. E. (2019). *Advanced Photon Source Upgrade Project Final Design Report.* Technical Report. Argonne National Laboratory, Argonne, IL, USA.

[bb13] Fusseis, F., Steeb, H., Xiao, X., Zhu, W., Butler, I. B., Elphick, S. & Mäder, U. (2014). *J. Synchrotron Rad.* **21**, 251–253. 10.1107/S1600577513026969 24365944

[bb14] Goodfellow, I., Bengio, Y. & Courville, A. (2016). *Deep Learning*, 1st ed., ch. 6, 9, 14. Cambridge: MIT Press.

[bb15] Hallot, M., Nikitin, V., Lebedev, O. I., Retoux, R., Troadec, D., De Andrade, V., Roussel, P. & Lethien, C. (2022). *Small*, **18**, 2107054. 10.1002/smll.202107054 35174974

[bb16] Huang, Z.-K. & Chau, K.-W. (2008). *Appl. Math. Comput.* **205**, 899–907.

[bb17] Iassonov, P., Gebrenegus, T. & Tuller, M. (2009). *Water Resour. Res.* **45**, w09415.

[bb18] Kang, W.-X., Yang, Q.-Q. & Liang, R.-P. (2009). *In*, pp. *Proceedings of the First International Workshop on Education Technology and Computer Science (ETCS 2009)*, 7–8 March 2009, Wuhan, Hubei, China, Vol. 2, pp. 703–707. IEEE.

[bb19] Kim, J. J., Lee, S. S., Fenter, P., Myneni, S. C. B., Nikitin, V. & Peters, C. A. (2023). *Environ. Sci. Technol.* **57**, 3104–3113. 10.1021/acs.est.2c07678 PMC9979612 36781166

[bb20] Lei, L. & Santamarina, J. (2018). *J. Geophys. Res. Solid Earth*, **123**, 2583–2596.

[bb21] Long, J., Shelhamer, E. & Darrell, T. (2015). *Proceedings of the IEEE Conference on Computer Vision and Pattern Recognition*, pp. 3431–3440. New York: IEEE.

[bb22] Nikitin, V. (2023). *J. Synchrotron Rad.* **30**, 179–191. 10.1107/S1600577522010311 PMC9814072 36601936

[bb23] Nikitin, V. V., Dugarov, G. A., Duchkov, A. A., Fokin, M. I., Drobchik, A. N., Shevchenko, P. D., De Carlo, F. & Mokso, R. (2020). *Mar. Petrol. Geol.* **115**, 104234.

[bb24] Nikitin, V. V., Fokin, M. I., Dugarov, G. A., Drobchik, A. N., Andrade, V. D., Shevchenko, P. D., Manakov, A. Y. & Duchkov, A. A. (2021). *Fuel*, **298**, 120699.

[bb25] Rezaei, F., Izadi, H., Memarian, H. & Baniassadi, M. (2019). *J. Petrol. Sci. Eng.* **177**, 518–527.

[bb26] Ronneberger, O., Fischer, P. & Brox, T. (2015). *18th International Conference on Medical Image Computing and Computer-Assisted Intervention (MICCAI 2015)*, 5–9 October 2015, Munich, Germany, pp. 234–241. Springer.

[bb27] Saxena, N., Hofmann, R., Alpak, F. O., Dietderich, J., Hunter, S. & Day-Stirrat, R. J. (2017). *Mar. Petrol. Geol.* **86**, 972–990.

[bb28] Sell, K., Saenger, E. H., Falenty, A., Chaouachi, M., Haberthür, D., Enzmann, F., Kuhs, W. F. & Kersten, M. (2016). *Solid Earth*, **7**, 1243–1258.

[bb29] Sinchuk, Y., Kibleur, P., Aelterman, J., Boone, M. N. & Van Paepegem, W. (2020). *Materials*, **13**, 936. 10.3390/ma13040936 PMC7079634 32093177

[bb30] Wang, J.-Q., Zhao, J.-F., Yang, M.-J., Li, Y.-H., Liu, W.-G. & Song, Y.-C. (2015). *Fuel*, **145**, 170–179.

[bb31] Zhang, Q.-B., Liu, K., Wu, G. & Zhao, J. (2022). *Handbook of Damage Mechanics: Nano to Macro Scale for Materials and Structures*, edited by G. Z. Voyiadjis, pp. 379–422. New York: Springer.

[bb32] Zhang, Y. & Duanquan, X. (2012). *J. Comput. Applic.* **32**, 134–136.

